# Efficacy and safety of anatomic resection versus nonanatomic resection in patients with hepatocellular carcinoma: A systemic review and meta-analysis

**DOI:** 10.1371/journal.pone.0186930

**Published:** 2017-10-26

**Authors:** Yifei Tan, Wei Zhang, Li Jiang, Jiayin Yang, Lunan Yan

**Affiliations:** Liver Transplantation Center, Department of Liver Surgery, West China Hospital of Sichuan University, Chengdu, Sichuan Province, China; Taipei Veterans General Hospital, TAIWAN

## Abstract

**Background:**

The surgical decision of performing anatomic resection (AR) or nonanatomic resection (NAR) in patients with hepatocellular carcinoma remains controversial. The aim of the current study is to conduct a meta-analysis on published results to compare surgical outcomes after AR and NAR.

**Methods:**

A comprehensive search of the Pubmed, Ovid-Medline, Embase, Cochrane library, and Science Citation indexes was performed. Overall and disease free survival (DFS), perioperative mortality and morbidity were the main outcomes. The meta-analysis was performed using Revman 5.3 statistical software, and the results are expressed as the relative risk (RR) or weighted mean differences with 95% of confidence intervals.

**Results:**

After application of the exclusion and inclusion criteria, 25 studies published between 1996~2015 that compared outcomes after AR and NAR in patients with HCC were identified. A total of 10216 patients were included in the meta-analysis, 4576 in the AR group and 5640 in the NAR group. Liver cirrhosis was found in 54.8% (range from 18.8% to 100%) of patients in the AR group and 67.8% (range from 34.3% to 100%) of patients in the NAR group, resulting in a RR of 0.45 (I2 = 18%, fixed model, 95% CI 0.39–0.52; Z = 10.31; P = <0.00001). The meta-analysis revealed a statistically significant 5-year survival (RR of 1.10, 95% CI 1.03–1.17; Z = 2.92, P = 0.004) and DFS (RR: 1.33, 95% CI 1.18–1.51; Z = 4.46, P <0.00001) advantage for patients undergoing AR resection compared to NAR. In regards to safety, no statistical significance was found in mortality and morbidity between the two groups. Eight studies including 1812 patients with small (<5 cm) solitary HCC indicated a better 5-year DFS in the AR group (41.4%) than in the NAR group (28.6%), with a RR of 1.32 (I2 = 42, fixed model, 95%CI: 1.15–1.52, Z = 3.86, P = 0.0001).

**Conclusion:**

The current study demonstrates better surgical outcomes after AR than NAR in patients with HCC. Therefore, AR is recommended in resectable HCC, especially with small (<5 cm) solitary tumours.

## Introduction

Hepatocellular carcinoma (HCC) is the one of the most common malignant cancers worldwide[1], and its incidence continues to rise because of various risk factors, particularly hepatitis induced cirrhosis and non-alcoholic steatohepatitis (NASH)[2–4]. In addition, HCC is the 3rd largest contributor to cancer related deaths, and its mortality rates could be as high as 43.2 per 100,000(Asians/Pacific Islanders)[5]. As with most solid tumours, curative resection of the liver is widely regarded as the first line therapy for HCC due to its acceptable mortality, morbidity and long term outcomes[6, 7]. Conventional limited resection, nonanatomic resection (NAR), is focused on achieving a non-tumoural liver parenchyma rim, without consideration of the Glisson’s pedicles[8, 9]s. Because of the underlying liver diseases of most patients with hepatocellular carcinoma, such as chronic hepatitis and cirrhosis, NAR is regarded to be useful for retaining as much liver parenchyma as possible[10]. This technique is extremely significant in patients with cirrhosis because the cirrhotic liver has a very limited capacity to regenerate[11], which is closely related to the long-term prognosis.

However, a tumour-free margin is not the only considerable factor in terms of recurrence, Kang et al[12] argued that the safety margin width could not provide a better local control rate and that tumour cells could easily spread through the portal venous system rather than only by adjacent diffusion. Anatomic liver resection (AR) is defined as resection of the neoplasm together with the portal vein related to the neoplasm and the corresponding hepatic territory[13]. Theoretically, AR is able to avoid intrahepatic metastasis and recurrence due to the invasion of tumour cells along portal veins and their intrasegmental branches[14, 15], and it is recommended as a feasible, safe, effective procedure for HCC[16]. However, AR needs to sacrifice a large amount of liver parenchyma to guarantee eradication of possible vascular invasions and daughter nodules and is therefore significantly unfavourable for treating a liver that has an underlying disease[17]. Furthermore, AR requires complex surgical procedures and highly precise real-time ultrasound guidance. In regard to long-term survival and recurrence-free survival, no clear evidence is available regarding the superiority of AR compared with NAR. Most of the previous studies that compared outcomes after AR with those after NAR used a heterogeneous group of patients that included patients with different stages of underlying liver disease and various tumour conditions. Some studies have reported the benefits of AR compared with NAR[7, 8, 18, 19], while other research has failed to obtain the same results[20–22], and some meta-analyses have also reported conflicting conclusions[23–25]. Therefore, the ideal treatment for HCC remains undetermined due to the contradictory nature of the published evidence. No randomized controlled trial is available, except a conference abstract[26], and this study is aimed at performing a meta-analysis comparing the main outcomes following AR and NAR in patients with hepatocellular carcinoma by using published observational trials.

## Methods

### Search strategy

A comprehensive search of the Pubmed, Ovid-Medline, Embase, Cochrane library, and Science Citation indexes was performed to retrieve studies published in English between January 1990 to 2015 (cut-off date Dec 4 2015) using the medical subheadings(MeSH) “Hepatocellular carcinoma” and “Hepatectomy”. The following words were used as key words to complete the search: “anatomic resection”, “nonanatomic resection”, “systemic resection”, “limited resection”, “partial resection”, and “wedged resection”. The abstracts of the articles obtained from the initial search were reviewed to identify relevant studies, and the references of the relevant articles were also used in an extensive backward search to ensure a systematic search. Reviews, case reports, letters, animal or in vitro studies, editorials or expert opinions, and conference abstracts were excluded in the search (S1 Table).

The following were the inclusion criteria of the meta-analysis: 1) clear definition and indication for AR and NAR for hepatocellular carcinoma; 2) comparison of AR and NAR as treatments for primary hepatocellular carcinoma; 3) report on the survival or DFS for a specific time after hepatectomy; and 4) if dual (or multiple) studies were reported by the same institution and/or authors, either the higher quality publication or more recent publication was included in the analysis.

The following were the exclusion criteria of the meta-analysis: 1) studies reported outcomes after AR or NAR alone without comparison; 2) studies reported on samples containing patients with tumour recurrence or liver metastases; and 3) we were unable to extract the outcomes needed to make a precise comparison from the published results. Any abstract that mentioned survival after liver resection or a performed comparison between anatomic and nonanatomic hepatectomy was assessed for inclusion. After screening the abstracts, the full text was read to confirm whether the article was should be included or not when the decision was not easily made on the basis of abstract as well as to find possible articles related to the subject.

**Fig 1:**
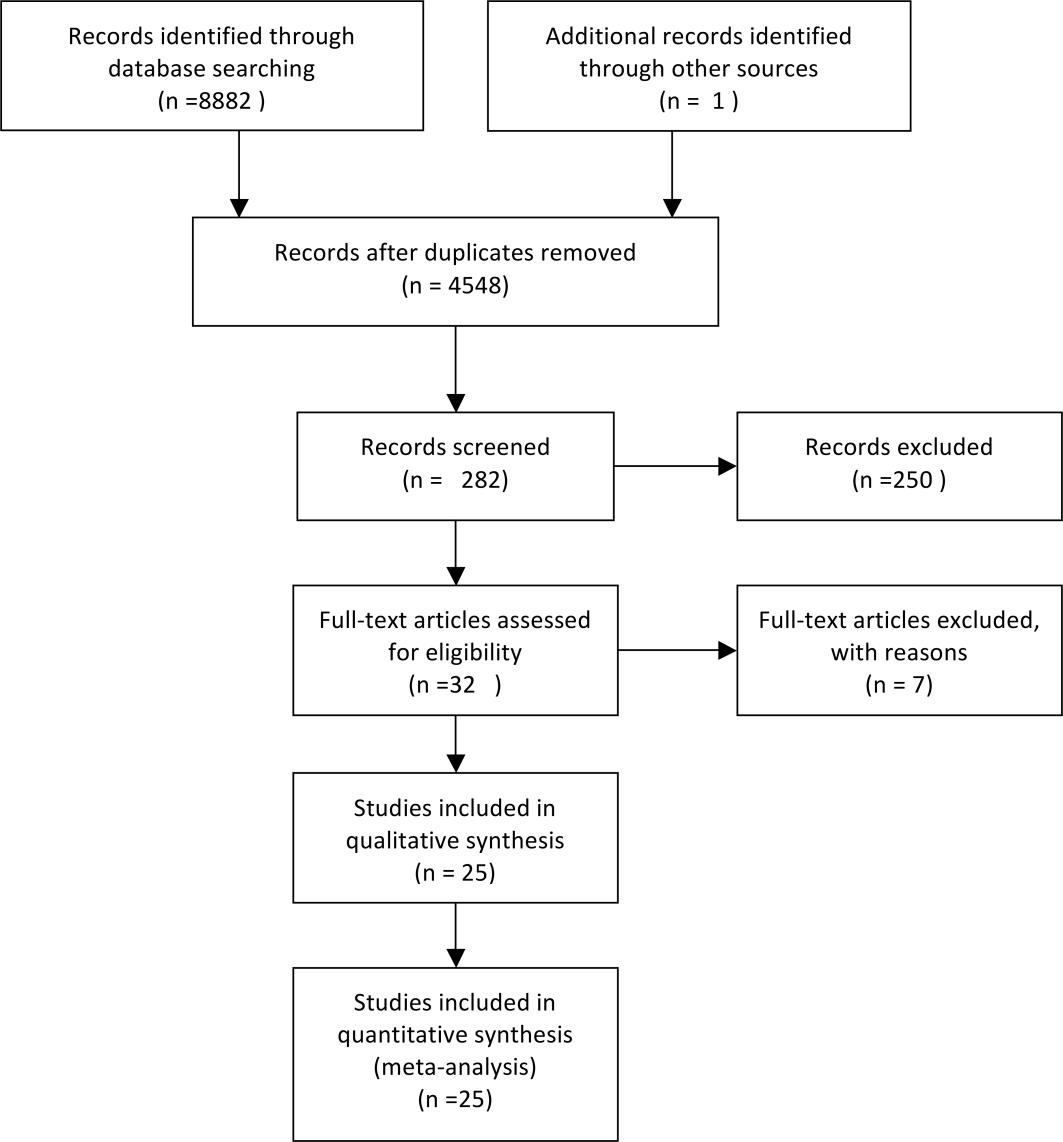
Flow diagram showing the search strategy along with the selection and screening processes for the eligible studies.

The literature search and assessment of the quality of articles were independently performed by two reviewers, YF, Tan and W, Zhang, and the quality of studies was assessed by means of the Newcastle–Ottawa Scale[27]. The quality of the studies was assessed by: patient selection, comparability of the study groups, and assessment of the outcome. Studies with high quality were those with more than 6 scores. Fig 1 depicts the search strategy in detail.

### Statistical methods

Overall survival and disease free survival at a certain times were the main outcomes that were assessed by the meta-analysis. The safety of the two types of resections, including perioperative mortality and morbidity, was regarded as a secondary main outcome. An estimate of the number of patients who survived for 5 years was calculated by multiplying the total number of patients in the anatomic and NAR subcategories included in the study by the corresponding 5-year OS estimate, and a similar procedure was performed to calculate the number of patients surviving without recurrence. The risk ratios (RRs) and 95% confidence intervals (CIs) were computed for the binary data, and the weighted mean differences with 95% (CI) were calculated for continuous data. Because of the large heterogeneity, original data were listed in the basic information table when continuous variables were reported as medians and ranges. The chi-squared test was used to explore heterogeneity with a significance level of P = 0.10, and a random-effect model was used when P<0.10; otherwise, a fixed-effect model was preferred[28]. A quantified description of heterogeneity was performed on the basis of I^2^ and a P value more than 0.10; I^2^ lower than 50% was defined as low heterogeneity. Funnel plots were used to explore publication bias. To address the heterogeneity and other issues of the current meta-analysis, subset analyses were performed, including patients with well compensated liver cirrhosis and single small tumours as well as studies with high quality. The Z-test was used to determine the overall effect, with the statistical significance of a two sided P value<0.05. Meta-analysis was performed using Review manager software (Version 5.3.5. Copenhagen: The Nordic Cochrane Centre, The Cochrane Collaboration, 2014.).

## Results

From the search with the Mesh terms and key words, 4548 studies were initially found, and 282 remained after title and abstract review. Reviews, case reports, letters, animal or in vitro studies, editorials or expert opinions, conference abstracts, and article reporting the outcomes of HCC after liver transplantation or non-resectional therapy or comparing resection therapy with transplantation/non-resectional therapy were excluded. Finally, 25 studies[1, 6–9, 12, 17–22, 29–41] published between 1996~2015 that compared the outcomes after AR and NAR in patients with HCC were identified. Three studies[1, 40, 41] analysed matched samples that contained the same number of patients selected from each group by performing propensity score matching to minimize the effect of potential confounders. Most studies were scored 6 or 7 (84.2%), and 13 studies (52.2%) were evaluated to have high quality (>6 scores).

### Patient characteristics

A total of 10216 patients from the 25 relevant studies were included in the meta-analysis, 4576 in the AR group and 5640 in the NAR group, and the number of eligible patients per study ranged from 46 to 5781. In terms of the whole analysis, 2609 (61.4%) patients presented with cirrhosis out of the 4274 patients who were reported to have cirrhosis or not. Similarly, 6361 (72.4%) patients were assessed as Child-Pugh A class for liver function out of 8780 patients, 932 (31.5%) patients were found to have hepatitis B out of 2957 patients, and 1868 patients (58.3%) tested positive for the hepatitis C antibody out of 3204 patients. The proportion of patients who had a microvascular invasion of the tumour was 27.2% (597 in 2197). Most studies had a mean or median follow-up of approximately 5 years. The important characteristics of these 25 studies are summarized in Table 1.

**Table 1:** Characteristics of the 25 studies included in the meta-analysis.

author	period	group	number	age	cirrhosis (%)	Child-A (%)	HBV (%)	HCV (%)	Size	ICG	AFP	MVI (%)	solitary (%)
Yamamoto(2001)	1990~1994	AR	90	–	38.9	–	–	–	2.9+1.0	–	–	21.1	–
	1990~1994	NAR	114	–	59.6	–	–	–	2.7+1.0	–	–	18.4	–
Regimbeau(2002)	1990–1996	AR	30	60 ± 11	100	100	–	–	3.0 ± 1.0	–	–	27	–
	1990–1996	NAR	34	61 ± 9	100	100	–	–	3.0 ± 1.0	–	–	26.5	–
Ziparo (2002)	1974–2000	AR	18	–	83.3	100	–	–	6.7(2.8–13.0)	–	–	–	–
	1974–2000	NAR	28	–	100	82.1	–	–	4.3(1.3–10.0)	–	–	–	–
Capussotti(2005)	1985–2001	AR	164	–	100	79.9	–	–	–	–	–	–	–
	1985–2001	NAR	52	–	100	73.1	–	–	–	–	–	–	–
Hasegawa (2005)	1994–2001	AR	156	64 (13–83)	32.1	85.9	25	62.2	3.5 (1.2–20.5)	12 (2–37)	23 (1–436,000)	–	–
	1994–2001	NAR	54	65 (16–79)	57.4	74.1	19	75.9	3.0 (1.2–17.0)	21 (5–63)	25 (1–49,388)	–	–
Kaibori(2006)	1992–2003	AR	34	65.1+7.4	29.4	79.4	–	100	4.1 ± 2.1	15.60+6.02	580+1332	67.6	64.7
	1992–2003	NAR	213	65.9+7.2	54	61	–	100	3.3 ± 2.3	20.34+9.52	927+8211	48.8	72.7
Cho (2007)	1998–2001	AR	99	55.9+9.5	55.6	92.9	–	20.2	3.5 ± 1.0	–	–	23.2	–
	1998–2001	NAR	69	57.4+8.9	72.5	91.3	–	15.9	3.1 ± 1.1	–	–	10.1	–
Wakai (2007)	1990–2004	AR	95	66 (29–80)	46.3	89.5	27.4	54.7	3.5 (1.2–17.0)	13.3 (3.0–43.0)	–	31.6	100
	1990–2004	NAR	63	64 (35–79)	66.7	77.8	15.9	60.3	3.0 (1.0–12.0)	17.4 (4.3–48.0)	–	12.7	100
Yamashita (2007)	1985–2004	AR	201	60+1	48.8	95	38	60	2.9 ± 0.1	16+0.6	476+149	–	–
	1985–2004	NAR	120	62+1	68.3	92.5	28	60	2.4 ± 0.1	22+1.0	315+96	–	–
Tanaka (2008)	1992–2005	AR	83	66 (41–77)	38.6	97.6	33.3	67.5	3.0 (1.1–12.0)	13.4 (4.5–37.9)	24 (1–20139)	–	80.1
	1992–2005	NAR	42	65 (41–80)	52.4	95.2	16.7	69	3.0 (0.8–9.0)	15.9 (5.8–38.2)	20 (3–102960)	–	73.4
Kobayashi (2008)	1990–2004	AR	106	65 (21–83)	23.6	96.2	23.6	62.3	3.0 (1.1–14.0)	15 (5–30.0)	–	29.2	100
	1990–2004	NAR	127	67 (33–88)	54.3	88.2	24.4	67.7	2.8 (1.0–14.5)	21 (2–58)	–	28.3	100
Eguchi (2008)	1994–2001	AR	2267	62.7+9.23	–	–	–	–	–	–	–	–	100
	1994–2001	NAR	3514	63.4+9.02	–	–	–	–	–	–	–	–	100
Nanashima (2008)	1994–2005	AR	49	65 ± 9	38.8	95.9	61.2	38.8	–	–	474	–	–
	1994–2005	NAR	64	64 ± 9	42.2	70.3	46.9	53.1	–	–	439	–	–
Ueno (2008)	1990–2004	AR	52	62 (28–85)	51.9	84.6	19.2	57.7	2.2 ± 0.6	13.6 ± 12.5	480 ± 1633	25	100
	1990–2004	NAR	64	63 (40–79)	67.2	71.9	18.7	73.4	2.1 ± 0.6	19.0 ± 9.9	178 ± 489	21.9	100
Kang (2010)	1998–2005	AR	146	52.3+9.8	54.8	–	–	–	2.8 ± 0.8	10.2+9.9	1114.8+4257.3	15.1	–
	1998–2005	NAR	21	51.2+9.8	76.2	–	–	–	2.7 ± 0.9	11.9+10.2	464.1+798.9	9.5	–
Yamazaki (2010)	1994–2007	AR	111	64.8 ± 11.1	55	99.1	18	70.3	3.1 ± 0.9	–	–	–	100
	1994–2007	NAR	98	66.0 ± 8.5	69.4	89.8	7.1	76.5	2.7 ± 1.1	–	–	–	100
Kamiyama (2010)	1990–2006	AR	169	–	27.2	98.8	–	34.9	–	15.7+8.69	–	32.5	100
	1990–2006	NAR	153	–	51	82.4	–	58.8	–	20.2+9.82	–	21.6	100
Dahiya (2010)	1983–2002	AR	159	–	100	–	–	–	–	–	–	–	–
	1983–2002	NAR	214	–	100	–	–	–	–	–	–	–	–
Tomimaru (2012)	1990–2008	AR	30	65 ± 8	40	100	83.3	76.7	2.2 ± 0.6	10.0 ± 3.5	565 ± 1584	10	–
	1990–2008	NAR	62	64 ± 8	48.4	100	83.9	37.1	2.1 ± 0.6	10.3 ± 2.8	205 ± 700	11.3	–
Sasaki (2013)	1990–2010	AR	30	62.0 (36–80)	–	90	26.7	50	–	–	–	–	100
	1990–2010	NAR	57	64.5 (35–80)	–	73.7	29.8	50.9	–	–	–	–	100
Fan (2013)	2005–2007	AR	81	–	–	–	–	–	–	–	–	–	–
	2005–2007	NAR	80	–	–	–	–	–	–	–	–	–	–
Kudo2014	2000–2012	AR	121	67.3+9.5	44	–	16.5	53	3.3+1.1	15.7+10.1	583+4118	–	96
		NAR	112	66.9+10.6	61	–	18.8	58	2.6+1.1	21.1+13.9	125+377	–	96
Cucchetti(2014)	2001–2010	AR	149	57 (48–66)	100	–	56.4	36.2	3.0 (2.0–4.0)	–	–	51.7	100
	2001–2010	NAR	149	56 (47–66)	100	–	57	35.6	3.0 (2.0–4.0)	–	–	51	100
Okamura(2014)	2002–2013	AR	64	71 (44–83)	12()	96.9	21.9	45.3	3.0(7–16.0)	16 (5–32)	8.7 (1.6–82,587)	7.8	100
	2002–2013	NAR	64	67 (39–83)	22()	100	21.9	53.1	2.5 (1.0–16.0)	17 (7–37)	11.8 (2.1–24,982)	17.2	100
Hirokawa (2015)	2001–2005	AR	72	69 (32–84)	36	93.1	15.3	65	3.0 (0.5–5.0)	13 (2–30)	14 (2–10,776)	0	–
	2001–2005	NAR	72	67 (30–86)	35	91.7	15.3	67	3.0 (1.0–5.0)	13 (5–35)	13 (1–239,599)	0	–

Abbreviations: Child-A: Child-Pugh A; HBV, hepatitis B virus infection; HCV, hepatitis C virus infection; ICG, indocyanine green retention rate; AFP, alpha-fetoprotein MVI, microvascular invasion

The patient characteristics were found to be significantly different in the AR and NAR groups. Liver cirrhosis was found in 54.8% of patients (ranged from 18.8% to 100%) in the AR group and 67.8% of patients (ranged from 34.3% to 100%) in the NAR group, resulting in a RR of 0.45 (I^2^ = 18%, fixed model, 95% CI 0.39–0.52; Z = 10.31; P = <0.00001. Concerning liver function, a marginally higher proportion of Child-Pugh class A patients was found in the AR (ranged from79.4% to 100%) group than in the NAR group (ranged from 61% to 100%), with a RR of 2.26 (I2 = 40%, random model, 95% CI 1.66–3.06; Z = 5.24, P<0.00001). The indocyanine green (ICG) retention rate at 15 minutes was considerably lower in the AR group than in the NAR group, with a pooled mean difference of -4.04 (random model, I2 = 91%, 95%CI:-6.27–1.80, Z = 3.54, P = 0.004). A higher prevalence of hepatitis B infection was found in the AR group (ranged from 15.0% to 61.2%) than in the NAR group (ranged from 7.1% to 57.0%), resulting in a RR of 1.28 (I^2^ = 15%, fixed model, 95%CI 1.08–1.51, Z = 2.82, P = 0.005), and patients in the AR group had a lower RR of hepatitis C infection of 0.77 compared to those in the NAR group (I^2^ = 31%, fixed model, 95% CI 0.66–0.89; Z = 3.38, P = 0.0007). Patients in the AR group (26.8%) had a slightly lower prevalence of microvascular invasion of tumour in comparison with patients in the NAR group (27.4%) resulting in a RR of 1.36 ((I^2^ = 33%, fixed model, 95%CI 1.10–1.69, Z = 2.81, P = 0.005)). Finally, the weighted mean tumour size was statistically larger in the AR group than in the NAR group, with a pooled mean difference of 0.34 (random model, I^2^ = 76%, 95%CI: 0.18–0.50, Z = 4.12, P<0.0001). Similarly, the mean resection margin was found to be larger in the AR group than in the NAR group, resulting in a MD of 0.60 (random model, I^2^ = 88%, 95%CI: 0.28–0.91, Z = 3.68, P = 0.002).

In summary, patients in the AR group were characterized by a lower prevalence of cirrhosis, HCV infection and microvascular invasion; better hepatic function; larger tumour size; and higher prevalence of HBV infection in comparison with patients in the NAR group.

### Meta-analysis of the main outcome measures

**Fig 2:**
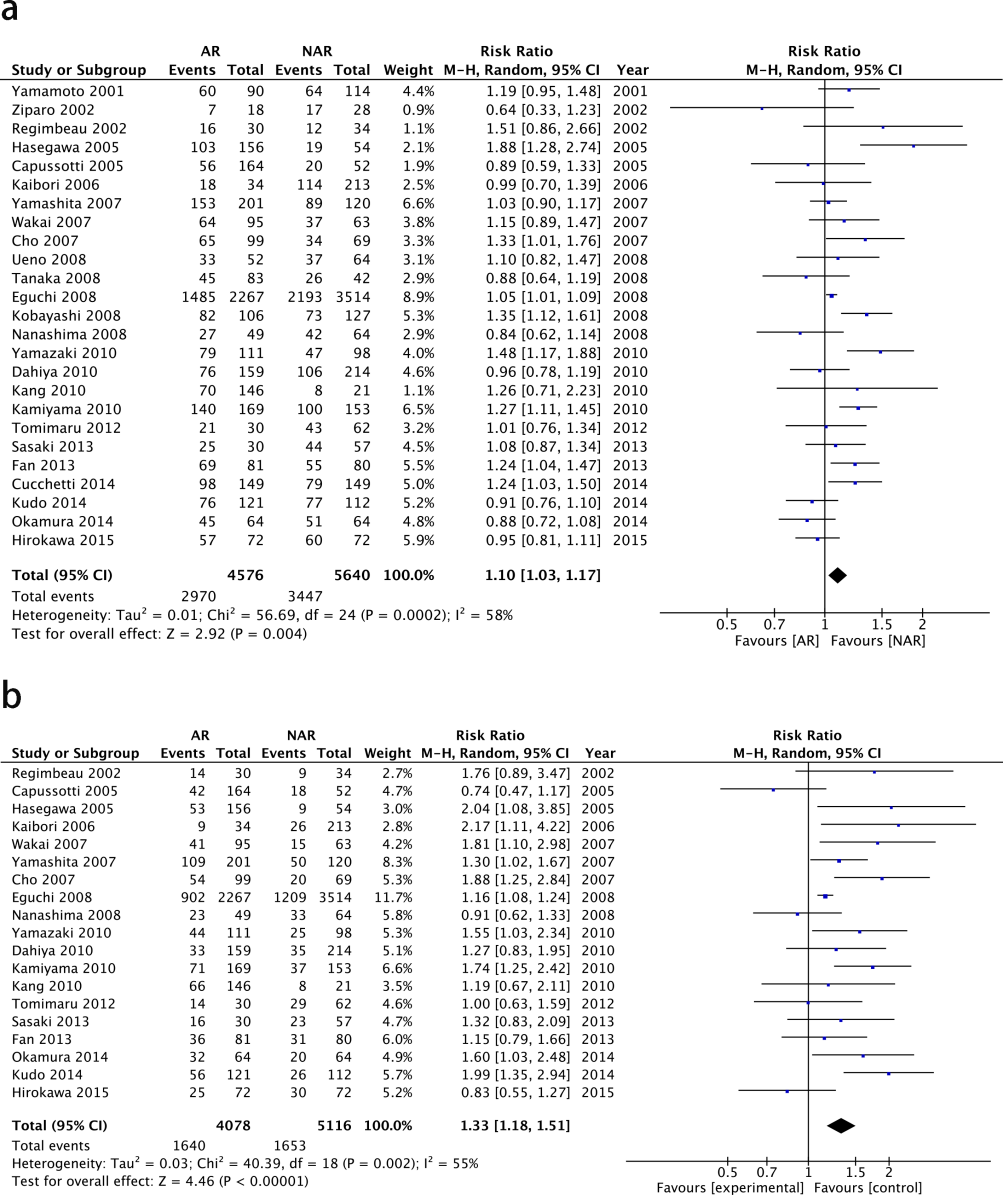
Forest plot of the results of the meta-analysis comparing long-term outcomes of the two groups. (a) 5-year overall survival of the AR group versus the NAR group. (b) 5-year disease free survival of the AR group versus the NAR group.

The surgical outcomes following AR and NAR in patients with HCC are shown in Table 2. The meta-analysis indicated that the 3-year overall survival of all patients with HCC was 79.1%. The 3-year postoperative OS rate was found to be slightly higher among patients in the AR group (81.5%) than patients in the NAR group (76.7%), without a statistically significant difference. The 5-year operative OS rates were 64.9% and 61.1%, respectively, of patients in the AR group and NAR group. The meta-analysis of the 25 studies revealed a statistically significant 5-year survival advantage for patients undergoing AR (2970 in 4576) compared to NAR (3447 in 5640), resulting in a RR of 1.10 (I^2^ = 58%, random model, 95% CI 1.03–1.17; Z = 2.92, P = 0.004). A summary of the data and forest plot to estimate the effect is shown in Fig 2. In regards to the DFS, 9194 patients were evaluated and had a 5-year DFS rate of 35.8%. Patients in the AR group (1640 in 4678) had a s significantly better disease free rate than patients in the NAR group (1653 in 5116), with a RR of 1.33 (I^2^ = 55%, fixed model, 95% CI 1.18–1.51; Z = 4.46, P <0.00001). No significant difference was found about the type of recurrence between the two groups, with a RR of 0.93 (95%CI:0.83–1.05,p = 0.24) for intrahepatic recurrence and a RR of 1.28 (95%CI:0.59–2.76, p = 0.53)for extrahepatic recurrence.

**Table 2:** Surgical outcomes following AR compared to NAR in patients with HCC.

author	group	number	blood loss	operation time	resection margin	hospital stay(d)	mortality	morbidity	3-Year survival	5-Year survival	5-Year DFS	NOS
Yamamoto (2001)	AR	90	–	–	–	–	1.1	–	–	67	–	6
	NAR	114	–	–	–	–	2.6	–	–	55.8	–	
Regimbeau (2002)	AR	30	844 ± 689	–	–	15±8	16.6	–	–	37.7	–	6
	NAR	34	670 ± 773	–	–	19 ± 13	3.5	–	–	61.1	–	
Ziparo (2002)	AR	18	–	–	–	–	7	47	–	54	45	7
	NAR	28	–	–	–	–	6	53	–	35	26	
Hasegawa (2005)	AR	156	574 (21–4830)	–	2 (0–11)	–	0	–	84	66	34	7
	NAR	54	560 (15–2699)	–	4 (0–55)	–	0	–	66	35	16	
Capussotti (2005)	AR	164	–	–	–	–	–	38.4	–	33.9	25.9	6
	NAR	52	–	–	–	–	–	38.5	–	39.3	34.8	
Kaibori (2006)	AR	34	1779+1688	325+114	–	24.8+32.2	2.9	23.5	58.3	52.5	27.8	7
	NAR	213	1414+1777	270+102	–	20.0+22.5	1.9	25.8	72.3	53.7	12.4	
Wakai (2007)	AR	95	813 (113–8715)	317 (140–900)	–	–	0	12.1	73.6	65.5	54.4	7
	NAR	63	590 (10–10205)	265 (100–660)	–	–	0	20.3	63.8	49.7	28.6	
Yamashita (2007)	AR	201	1353+83	303+7	9+0.8	28+2	2	22	–	67	43	7
	NAR	120	993+80	263+8	5+0.5	25+2	6	25	–	59	24	
Cho (2007)	AR	99	–	–	1.2+1.0	29.1+18.3		16	–	76	54	7
	NAR	69	–	–	0.9+0.7	26.7+14.0		13	–	74	42	
Ueno (2008)	AR	52	1609 ± 1391	–	1.7± 1.6	–	–	–	–	77.3		6
	NAR	64	1224 ± 1290	–	0.6± 0.6	–	–	–	–	57.5		
Eguchi (2008)	AR	2267	–	–	–	–	0.71		–	65.5	39.8	6
	NAR	3514	–	–	–	–	0.86		–	62.4	34.4	
Kobayashi (2008)	AR	106	–	–	2 (0–20)		0	17	70	54		7
	NAR	127	–	–	4 (0–40)		0	21	91	61		
Nanashima (2008)	AR	49	680 ± 569	–	–	–	–	–	–	63		6
	NAR	64	348 ± 456	–	–	–	–	–	–	58		
Tanaka (2008)	AR	83		425 (105–787)	0.6 (0–6.0)	15 (8–85)	–	26.5	–	55	46	6
	NAR	42		406 (178–725)	0.6 (0–3.8)	16 (8–47)	–	37.5	–	66	51	
Kamiyama (2010)	AR	169	880.7+1245.2	–	–	–	–	–	–	83	41.8	6
	NAR	153	742.9+719.7	–	–	–	–	–	–	65.3	24.5	
Kang (2010)	AR	146	833.3+681.7	251.1+80.0	2.0+1.4	–	–	17.8	–	48	45	7
	NAR	21	716.7+396.1	211.9+72.9	0.8+0.6	–	–	4.8	–	40	40	
Yamazaki (2010)	AR	111	1266 ± 1062	–	0.9± 0.8	–	–	–	–	47.5	20.9	6
	NAR	98	842 ± 932	–	0.7+0.6	–	–	–	–	49.4	16.4	
Dahiya (2010)	AR	159	–	–	–	–	1.8	46	79	71	40	7
	NAR	214	–	–	–	–	0	42	71	48	25	
Tomimaru (2012)	AR	30	1112 ± 809	253±78	–	20±13	0	30	81.3	68.8	46.2	7
	NAR	62	756 ± 702	213 ± 59	–	19 ± 10	0	16.1	86.2	69.6	47.5	
Fan (2013)	AR	81	–	–	–	–	–	–	98	85	44.4	5
	NAR	80	–	–	–	–	–	–	81	69	38.8	
Sasaki (2013)	AR	30	216 (5–790)	–	0.55 (0–3)	–	1 ()	–	96	82.8	52.9	6
	NAR	57	123 (5–942)	–	0.5 (0–2.0)	–	1 ()	–	84.1	77	40.7	
Cucchetti (2014)	AR	149	–	–	–	–	–	–	83.3	65.8	37.2	8
	NAR	149	–	–	–	–	–	–	66.8	52.9	32.2	
Kudo (2014)	AR	121	–	–	–	–	–	–	78	63	46	6
	NAR	112	–	–	–	–	–	–	82	69	23	
Okamura (2014)	AR	64	551 (76–3,225)	271 (133–575)	0.7 (0–4.2)	11 (7–35)	0	–	91.2	71	50	8
	NAR	64	465 (12–2,569)	229 (83–619)	0.7 (0–2.5)	11 (5–57)	0	–	90.1	79.7	31.9	
Hirokawa (2015)	AR	72	715 (50–5,100)	308 (75–628)	–	19 (10–104)	–	24	86	79	35	8
	NAR	72	373 (10–2,110)	222 (110–465)	–	16 (9–54)	–	10	89	84	41	

**Fig 3:**
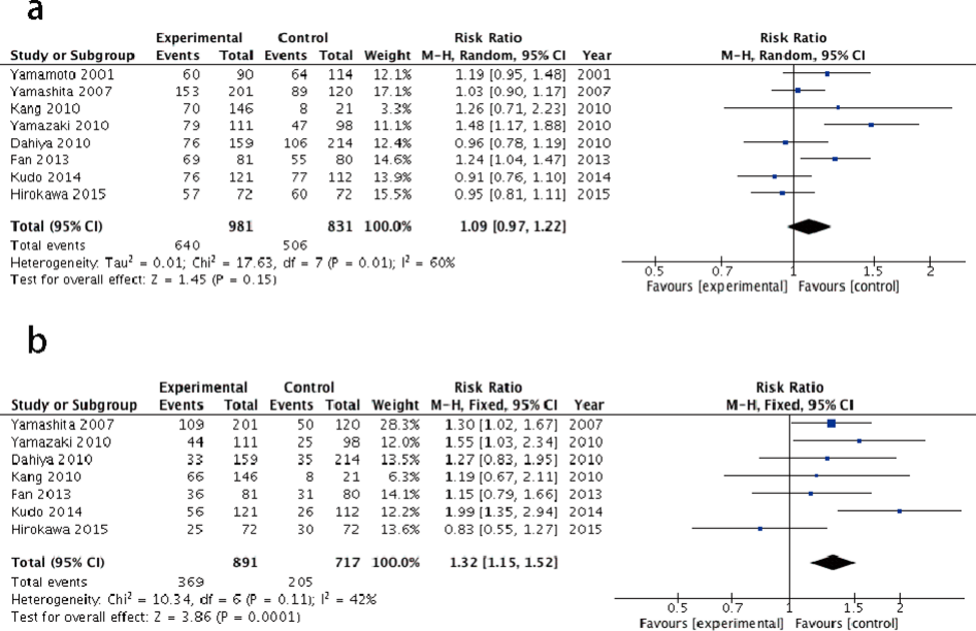
Forest plot of the results of the meta-analysis comparing long-term outcomes of the two groups in small solitary HCC. (a) 5-year overall survival of the AR group versus the NAR group. (b) 5-year disease free survival of the AR group versus the NAR group.

Eight studies including 1812 patients with small (<5 cm) solitary HCC had a better 5-year DFS in the AR group (41.4%) than in the NAR group (28.6%), with a RR of 1.32 (I^2^ = 42, fixed model, 95%CI: 1.15–1.52, Z = 3.86, P = 0.0001). However, no significant difference was observed in the 5-year OS between the two groups (RR = 1.09, fixed model, 95%CI: 0.97–1.22, Z = 1.45, P = 0.15). A summary of the data and forest plot to estimate the effect is shown in Fig 3.

In regards to safety and recovery, patients in the AR group (0.87%) had a lower mean 30 day mortality than the NAR group (9.91%), but the result was not statistically significant (RR = 0.93, 95CI:53%-1.47, Z = 0.32, P = 0.75). Patients in the AR group had a morbidity ranging from 12.11% to 47%, whereas patients in the NAR group had a similar morbidity, ranging from 10.0% to 53.0%, resulting in a RR of 1.04 (I^2^ = 21, fixed model, 95%CI:0.88–1.21; Z = 0.43, P = 0.67).

Patients who underwent AR suffered more blood loss during the operation than those in the NAR group, with a weighted mean difference of 303.95 (random model, I^2^ = 25%, 95%CI: 226.13–381.71, Z = 7.66, P<0.00001). Similarly, the mean time of surgery, which was closely correlated with blood loss, was statistically significantly longer in the AR group than in the NAR group (fixed model, I^2^ = 0%, MD = 40.02, 95%CI:38.30–41.75, Z = 45.57, P<0.00001). However, there was no statistically significant difference in the days of hospital days between the two groups, though there was a trend towards a longer hospital stay period following AR compared to NAR (AR vs. NAR, I^2^ = 47%, MD = 1.53, 95% CI: 1.07–4.13; Z = 1.16, P = 0.250).

## Discussion

Several treatment options are available to patients with hepatocellular carcinoma, and the ideal option is determined based on the tumour burden and underlying liver disease, of which cirrhosis is the most important. Liver resection, including anatomic or nonanatomic resection, remains the main therapy for HCC patients, while liver transplantation is well established in some developed countries[42]. Both AR and NAR have own advantages and limitations. AR removes both the tumour and potential pre-cancerous liver parenchyma, but with an increased risk of liver failure after surgery, which is less commonly seen in patients following NAR[22]. Whether AR is superior to NAR remains undetermined due to the heterogeneous groups used in previous studies, in which patients had different tumour burdens and underlying liver disease. The result of the present meta-analysis, which includes 25 studies, shows a statistically significant improvement in both the 5-year overall survival and disease free survival rates in patients who received AR compared to those who received NAR, though this difference was not observed at 3 years after liver resection. This superiority was shown in 14 out of 25 studies, while 7 studies failed to find a difference in outcomes between the two types of liver resection. Five studies[7, 8, 19, 29, 32] revealed AR to be an independent favourable factor for long-term outcomes (OS and/or DFS), and 4 studies[20–22, 36] drew the opposite conclusion. Slightly better perioperative mortality and morbidity were found in patients following AR, though the difference was not statistically significant. Most of the studies did not find any difference in terms of safety between the two groups except Hirokawa, F. et al[41], who argued that AR might lead to worse short-term outcomes in patients with a single tumour <3 cm in diameter.

With the advances in surgical techniques and perioperative care, the outcomes of HCC after curative liver resection have been steadily improving. However, long-term survival/recurrence free survival remains unsatisfactory because of the high incidence of recurrence. Previous studies have demonstrated two types of recurrence, intrahepatic metastasis and multicentric tumours[43, 44]. Intrahepatic metastasis mainly occurs in the early phase, whereas multicentric tumours usually occur in the late phase after hepatectomy for HCC.

Local recurrence is defined as a tumour within 2 cm of the surgical margin or in the segment of the initial tumour[45] / in the same section where the primary tumour was located[9]. As cancer spreads via the portal venous system, local recurrence is thought to be due to residual intrahepatic metastatic foci adjacent to the primary HCC[34]. Cancer spreads via the portal venous system, which is cannot be detected before or during surgery. Several studies[7, 8, 19, 29, 38, 39] have indicated the superiority of AR for the possible eradication of minute intrahepatic metastatic foci by resecting the tumour-bearing portal branches and corresponding liver parenchyma. The present meta-analysis has also revealed an improvement of both OS and DFS in patients who received AR compared to those who received NAR.

However, patients in the AR group also presented with a lower prevalence of cirrhosis and HCV infection as well as better liver function. In regards to liver cirrhosis, which has been shown to be an independent factor for HCC incidence and prognosis[46], four of the studies[1, 6, 9, 30] included in the current meta-analysis focused on selecting patients with cirrhosis, and a subgroup analysis revealed a better OS and DFS in patients who underwent AR compared to those who underwent NAR, but failed to find a statistically significant difference. Within these 4 studies, Regimbeau et al.[9] reported higher long term OS and DFS of the AR group and also found that there was less recurrence following AR compared to NAR, including overall recurrence (39% vs. 66%) and local recurrence (10% vs. 50%), but no significant difference was observed regarding the surgical treatment for recurrence between two groups (33% vs. 36%). Cucchetti et al[1] obtained a similar result in a population of 543 patients, though NAR patients suffered from worse hepatic dysfunction and more frequent HBsAg positive serology. However, these improvements of overall and disease free survival were not found in Dahiya’s[6] study, and Capussotti et.al[30] drew an opposite conclusion: a decrease in overall survival and recurrence free survival in patients following AR. Some studies have shown that there are some benefits of AR in selected cirrhosis patients with HCC compared to NAR, but the number of studies and patients might be too small to draw the conclusion that AR is superior to NAR in selected cirrhotic patients with HCC. Considering the limited number of patients, it is quite possible that this statistical insignificance will change with an increase in sample size.

Other researchers found better long-term outcomes, but not an independent prognosis factor, after AR in HCC patients, suggesting that a better liver function or less HCV infection could partly explain the better outcomes in the AR group, but as many as 5 different studies reported AR to be a favourable independent prognostic factor, which means that AR was a significant favourable factor related to OS and RFS by not only univariate analysis but also by multivariate analysis. These studies included the following different types of patients: tumours in T1-T2[32], single with a small size[7, 29], and meeting the Milan criteria[19]. It is widely believed that AR could theoretically eradicate the tumour-bearing portal system by completely removing the hepatic segment fed by the same portal veins, and therefore, the survival benefits of AR might be partly due to the better clearance of venous tumour thrombi within the adjacent liver[32] because even a small tumour could invade the portal vein and form a tumour thrombus[47]. However, AR or NAR had no significant influence on late recurrence after resection for HCC, which was mainly ascribed to multicentric occurrence[29, 34].

With the increasing awareness of personal health and rapid development of diagnostic approaches, especially radiologic imaging techniques, patients with small solitary HCC and well-preserved or acceptable liver function are currently more often diagnosed[12]. Eight studies[6, 7, 12, 17, 29, 33, 38, 41] included patients with small and single HCC in the current meta-analysis, and it was shown that AR conferred a remarkable improvement on 5-year disease free survival compared to NAR(41.4% vs. 28.6%) in patients with small solitary HCC. Actually, it has been shown that surveillance, which is aimed at early detection of HCC so that a better and more efficient therapy would be available, is helpful to make an early diagnosis, resulting in a significant improvement of long-term outcomes[48]. Because intrahepatic recurrence is the most frequent mode of HCC recurrence[9, 34, 45], which is thought to be caused by metastasis via the portal vein, this difference, in regard to recurrence free survival, might be explained by the ability of AR to eradicate intrahepatic metastasis confined to tumour-bearing portal tributaries, which poses a smaller risk of locoregional tumour progression than limited resection. Another important model of recurrence is multicentric tumours, and it is impossible to distinguish intrahepatic metastasis and multicentric tumours in multiple tumour cases, which is thought to be a reason why the recurrence free survival rate gradually decreases after surgery because AR is not able to prevent multicentric carcinogenesis.

It is widely believed that microvascular invasion (MVI) is a key factor in HCC recurrence, and Fan et al. [38] identified AFP as a unique predictor of MVI. They also found that AR achieved better survival outcomes over nonantomic resection when AFP>100 ug/L. Similar results were found by Kudo et al[17]. Other studies[1, 7] have indicated that AR has a certain prophylactic potential for intrahepatic metastases, especially for relatively small, histologically advanced (moderately or poorly differentiation) single HCCs larger than 2 cm in diameter; AR has also been recommended by a Japanese nationwide survey[18] including 5871 patients with HCC. However, AR has not been shown to make difference for HCC<2 cm,[7, 18], and Hiroka et al. [41]even reported that AR was more likely to lead a worse short-term outcome when tumours were smaller than 3 cm, including a significantly increased operation time, blood loss and transfusion, complications, and postoperative hospital stay. In terms of OS, no significant difference was found between the two groups, with a 5-year OS of 65.2% and 60.9%, respectively. Interestingly, early research[29] reported an OS improvement in patients following AR, but later studies failed to find a statistically significant difference between the two groups. This finding might be explained by the development of treatment for recurrence and the appearance of new techniques, such as radiofrequency ablation (RFA), transcatheter arterial chemoembolization (TACE) and percutaneous ethanol injection. In addition, AR has become a safer and easier procedure to perform with the help of modern techniques, especially continuous ultrasound monitoring of the liver[49]. Therefore, AR is recommended for patients with relatively small (2–5 cm) solitary moderately or poorly differentiated HCC, particularly when AFP>100 ug/L or in those who present with MVI. However, NAR might be a better choice for patients with better differentiated HCC smaller than 3 cm, but with worse liver damage, to prevent a postoperative liver insufficiency.

The most important limitation of the present meta-analysis is the lack of RCTs, though we included as many as 25 cohort studies. One RCT[26], which was found as a congress abstract, indicated a superior 1-year DFS in the AR group, and AR was confirmed to be an independent favourable factor of DFS by multivariate analysis, but specific data were not available. For nonrandomized trials, they might help improve the precision by including selected patients, and a propensity score matching process is suggested to reduce the bias caused by the clinicopathological factors, including demographic and clinical characteristics and tumour-related factors. Better designed studies are crucial to address this controversial issue regarding the long-term outcomes of the two main types of resection to provide feasible guidance to patients with HCC.

AR could lead to better overall survival and disease free survival in most patients with hepatocellular carcinoma, especially for small solitary tumours. However, NAR is recommended in patients with better differentiated HCC smaller than 3 cm, but with worse liver damage. A well-designed RCT is needed to confirm the superiority of AR compared to NAR in HCC patients.

## Supporting information

S1 Table PRISMA 2009 checklist for the meta-analysis. (DOC)
